# A Plastid-Bound Ankyrin Repeat Protein Controls Gametophyte and Early Embryo Development in *Arabidopsis thaliana*

**DOI:** 10.3389/fpls.2022.767339

**Published:** 2022-03-08

**Authors:** Katarína Kulichová, Janto Pieters, Vinod Kumar, David Honys, Said Hafidh

**Affiliations:** ^1^Laboratory of Pollen Biology, Institute of Experimental Botany of the Czech Academy of Sciences, Prague, Czechia; ^2^Department of Plant Experimental Biology, Faculty of Science, Charles University, Prague, Czechia

**Keywords:** proplastid, pollen, embryo development, pollen tube reception, fertilization

## Abstract

Proplastids are essential precursors for multi-fate plastid biogenesis, including chloroplast differentiation, a powerhouse for photosynthesis in plants. Arabidopsis ankyrin repeat protein (AKRP, AT5G66055) is a plastid-localized protein with a putative function in plastid differentiation and morphogenesis. Loss of function of *akrp* leads to embryo developmental arrest. Whether AKRP is critical pre-fertilization has remained unresolved. Here, using reverse genetics, we report a new allele, *akrp-3*, that exhibited a reduced frequency of mutant embryos (<13%) compared to previously reported alleles. *akrp-3* affected both male and female gametophytes resulting in reduced viability, incompetence in pollen tube attraction, altered gametic cell fate, and embryo arrest that were depleted of chlorophyll. AKRP is widely expressed, and the AKRP-GFP fusion localized to plastids of both gametophytes, in isolated chloroplast and co-localized with a plastid marker in pollen and pollen tubes. Cell-type-specific complementation of *akrp-3* hinted at the developmental timing at which AKRP might play an essential role. Our findings provide a plausible insight into the crucial role of AKRP in the differentiation of both gametophytes and coupling embryo development with chlorophyll synthesis.

## Introduction

Proplastids are essential organelles for plant life cycle; they provide nutrients and monomers for energy, act as a precursor for chloroplast differentiation, and function in biosynthesis and storage of pigments, hormones, starch, fats, proteins, and terpenes. During embryo development, proplastids differentiate into chloroplasts from the globular stage, and later, during seed maturation, differentiate into storage plastids, elaioplasts (Demarsy et al., 2012; Allorent et al., 2013; reviewed in Liebers et al., 2017). Many plastid genes are, thus, critical for early plant development, and mutations often result in embryo lethality (Tzafrir et al., 2004; Meinke et al., 2008; Ajjawi et al., 2010; Myouga et al., 2010, 2013; Bryant et al., 2011). Defects in plastid function might also manifest prior to embryo development in the male or female gametophyte. Energy production in mature pollen and growing pollen tubes (PTs) is mainly supplied from mitochondrial respiration and ATP production; nevertheless, glycolysis within plastids is also a contributing source of energy (reviewed in Selinski and Scheibe, 2014). Accumulation of starch during pollen maturation is, therefore, critical, as it serves later as an energy source. Knockout mutants in enzymes catalyzing the regeneration of NAD + during glycolysis, *gapcp1* and *gapcp2*, are sterile males (Muñoz-Bertomeu et al., 2010). The double mutant genes, *gapcp1* and *gapcp2*, exhibit defects in pollen morphology and viability in addition to having a disorganized tapetal layer. Several other mutants involved in glycolytic processes and maintaining redox homeostasis with defects in gametophyte and embryo development have been described (Prabhakar et al., 2010; Chen et al., 2011; Zhao and Assmann, 2011; reviewed in Selinski and Scheibe, 2014).

Almost all types of plastids are present during pollen development in several layers of the microsporangium and are essential in the formation of functional male gametophytes (Clément and Pacini, 2001; Hafidh and Honys, 2021). They play a supportive function to other cells as in the case of tapetum and the vegetative cell, but proplastids in sperm cells play a direct role in reproductive events through biparental cytoplasmic inheritance to the newly formed zygote. In angiosperms, only proplastids and amyloplasts are present from meiocytes to pollen maturity. Amyloplasts differentiated from proplastids in microspores are preceded by vacuolization as pollen matures (Pacini, 1994). In contrast, proplastids from tapetum undergo division during the early stages of microsporogenesis and differentiate into elaioplasts (Dickinson, 1973; Pacini and Juniper, 1979). Both tapetosomes and elaioplasts generate tapetal lipids that are secreted into the locule to form tryphine when the tapetal plasma membrane breaks down (Dickinson and Lewis, 1973). They also constitute oleosins and triacylglycerols, which later reach the coat of pollen and form a major lipid component (Ting et al., 1998).

Arabidopsis *EMB2036*, an ankyrin repeat protein (AKRP), is a single copy gene with five ankyrin repeats (Zhang et al., 1992). Ankyrin repeat-containing domains (ANK) are described as protein-protein interaction domains found in viruses, archea, bacteria, and eukaryotes. Ankyrin repeats have been found in numerous proteins with functions, such as cell signaling, cytoskeleton integrity, transcription and cell–cycle regulation, inflammatory response, development, and various transport pathways. No enzymatic function has been detected in any ankyrin repeat-containing proteins (reviewed in Mosavi et al., 2004). In Arabidopsis, there are 105 ANK-containing proteins, but only a few were described functionally (reviewed in Becerra et al., 2004). Two ankyrin repeat-containing plastid-targeted proteins, NPR1-like protein 3 and NPR1-like protein 4, are receptors for salicylic acid and are important for basal defense against pathogens (reviewed in Kuai et al., 2015). A mitochondrial protein, ANK6, is an example with function in gametophyte development and male-female gamete recognition during double fertilization (Yu et al., 2010). An *AKRP* expression was reported to be developmentally and lightly regulated. Plants transformed with sense or antisense constructs exhibited a chlorotic phenotype caused by a loss of chloroplast ultrastructure and lower amounts of chlorophylls and carotenoids (Zhang et al., 1992). Interestingly, the expression of selected photosynthesis-related genes was not affected in AKRP-deficient plants (Zhang et al., 1994). An AKRP interacted through ankyrin domains with a sequentially and functionally similar protein, EMB506 (Albert et al., 1999). Two predicted splice isoforms of *AKRP* were experimentally confirmed to be expressed (Garcion et al., 2006). Based on the observed mutant phenotype, AKRP was predicted to function in plastid differentiation. A recent study described a mechanistic role of AKRP (STT2) and EMB506 (STT1) as essential in the sorting of chloroplast twin-arginine translocation (cpTat) pathway proteins to thylakoid membranes (Ouyang et al., 2020). In this study, we have characterized a previously unknown gametophytic defect identified in a new T-DNA insertion allele of *akrp-3* that was absent in other reported *emb2036* alleles. Using gametophytic promoters, we provide a potential time window for AKRP function, pre- and post-fertilization.

## Results

### Isolation of Novel *akrp* Allele

**FIGURE 1:**
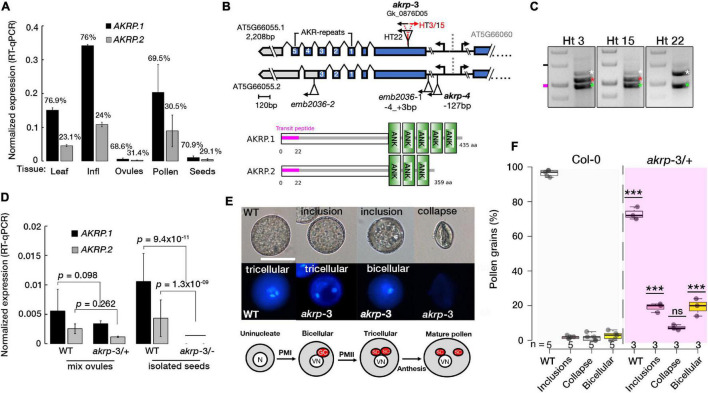
The *akrp-3* allele exhibits abnormal pollen maturation and loss of gametic cell fate. **(A)** Quantification of AKRP splice isoform expression by RT-qPCR. Percentages represent the relative abundance of each isoform in respective tissues. **(B)** Gene and protein models of two AKRP isoforms with the position of *akrp* T-DNA insertions and ankyrin repeats (ANK). Alignment of the isoforms is presented in Supplementary Figure S1. **(C)** PCR genotyping of *akrp*-3 (Gk_0876D05) revealed two groups of segregating heterozygous; with three bands (HT3/15) and those with two bands (HT22), wild-type band (white asterisks), mutant (green asterisks) and a second T-DNA band (red asterisks) 6 bp apart. **(D)** Estimation of knockdown of both isoforms in *akrp*-3 mixed ovules and isolated homozygous *akrp*-3 seeds. **(E)** Abnormal pollen development observed in *akrp-3/* + includes segregant WT pollen, reduced size pollen grains with cytoplasmic inclusion bodies with a single undivided generative nucleus (binucleate pollen) or with two sperm cells, and totally collapsed pollen grains with no DNA content. Below, a schematic of a normal pollen maturation. The N/VN are nuclei of the microspore and vegetative cell, GC/SC are the products of pollen mitosis I and II, the generative and the sperm cells, respectively. **(F)** Frequency of pollen phenotype observed in panel **(E)**. Statistical evaluation was performed using one-way ANOVA with Tukey’s multiple comparisons test, with “n”-representing individual M3 plants assessed. ***p < 0.0001, ns, not significant. *N* = 12,463 pollen grains, scale bar = 20 μm.

Two previous embryo-lethal alleles of *AKRP*, *emb2036-1* and *emb2036-2*, were reported to show 25% of pale mutant homozygous seeds containing embryos arrested at globular stage but exhibited no gametophytic defects (Garcion et al., 2006; Meinke et al., 2008). Public RNA-seq data suggest that AKRP transcripts are higher in microspores but are drastically reduced by pollen maturity, and were detected in isolated egg cell of Arabidopsis thaliana (Julca et al., 2021). As a single copy gene, the lack of gametophytic defect was puzzling. We, therefore, performed RT-qPCR measurements and verified that AKRP is broadly expressed and exhibits variation in abundance among sporophytic and gametophytic tissues with the highest expression in reproductive tissues (Figure 1A). Two splice variants are predicted to arise from alternative splicing of an *AKRP* single locus, with the shorter isoform lacking the last two ankyrin repeats (Figure 1B and Supplementary Figure S1). The RT-qPCR revealed that, on average, the longer isoform is >2.5-fold more abundant in both sporophytic and reproductive tissues (Figure 1A). We fused a 997bp putative AKRP promoter fragment with a beta-glucuronidase enzyme (GUS) and detected a promoter activity throughout development, with the strongest GUS staining specifically in the early stages of pollen development in ovules and the embryo, as well as in mature seeds (Supplementary Figure S2). Therefore, we screened numerous Gabi-kat T-DNA collections (^1^ Kleinboelting et al., 2012) with putative insertion in the *AKRP* locus and isolated a new allele, *akrp-3* (Gk_0876D05), which exhibited both pollen and female gametophytic defects as well as a similar embryo-lethal phenotype but in a reduced frequency. We confirmed the position of insertion by Sanger sequencing, which revealed T-DNA insertion between 609 and 624 bp of exon 1 (Figure 1B). No homozygous individuals were recovered, confirming a gametophytic or embryo lethality. However, among segregating heterozygous individuals, some contained the canonical wild type and a mutant T-DNA band (Ht22), and others had an extra band (Ht3 and HT15) that we confirmed by sequencing as a back-to-back T-DNA inserted 6 bp away from the first insertion (Figures 1B,C). Both types originated from a common parent stock; therefore, we assumed that they were identified and merged as a single event at the stock center. No significant phenotypic variations were observed between plants with single T-DNA or those with back-to-back T-DNA insertion genotypes, however, were possible we present both types separately. We quantified the expression of both *AKRP* isoforms by RT-qPCR and verified their downregulation in *akrp-3/*+ heterozygous ovules and complete knockout in isolated *akrp-3* homozygous mutant seeds (Figure 1D).

### Loss of Function of *akrp* Produced Abnormally Developed Pollen Without Gametic Cell Fate

In pollen, multiple aberrant phenotypes were identified *akrp*-3/+ plants. Among the aberrant pollen phenotype they include underdeveloped smaller pollen grains with cytoplasmic inclusion bodies, bi-nucleate pollen that failed to undergo the second pollen mitotic division (PMII) of the generative cell to produce two sperm cells, and the most severe was the appearance of aborted pollen grains (Figure 1E). No similar phenotypes were observed in segregating wild-type progenies or in screening of the original *emb*2036-1 allele (Figure 1B). The screening of an independent allele, *akrp-4*, with an insertion at *AKRP*5′UTR, did not exhibit any aberrant phenotypes. To rule out a T-DNA effect on a neighboring gene, AT5G66060, we screened three T-DNA alleles belonging to the AT5G66060 locus. None of the AT5G66060 alleles recapitulated the *akrp*-3 phenotype, which strongly supports that the observed phenotype is linked to *akrp*-3 loss of function.

To better understand the nature of the *akrp-3/*+ gametophytic defect, multiple assays were performed to assess viability and pollen fitness. Alexander staining revealed fully viable pollen grains alongside partially stained pollen grains containing cytoplasmic inclusion bodies, whereas *in vitro* germination showed slight reduction in *akrp*-3/+ pollen germination (Supplementary Figures S3a–c). The PT length appeared similar whether *in vivo*, semi-*in vivo* or *in vitro* (Supplementary Figures S3d,e). However, *akrp*-3 PT length perturbation could be masked by the mixture with wild-type PTs, as they are not phenotypically differentiated. The failure of *akrp*-3 generative cell to undergo PMII and appearance of inclusion bodies in the *akrp*-3 pollen implied possible pollen developmental perturbation. We therefore investigated if *akrp* gametic cell fate is correctly specified. We crossed multiple gametophyte cell-specific markers into *akrp-3/*+ plants. These included an egg cell marker (pEC1.1-H2B-mRFP), a sperm cell marker (pHTR10-HTR10:RFP), and vegetative cell markers (pLat52-GFP and pLat52-GUS). The screening of *akrp-3/*+ plants homozygous for cell fate markers revealed that pEC1.1-H2B-mRFP egg cell expression was reduced to half of the ovules compared to that of wild-type control (35.9%, *n* = 1733), indicative of an effect on *akrp*-3 ovule egg cell differentiation (Supplementary Figures S3f,g). Similarly, the expressions of pLat52-GFP, pLat52-GUS, and pHTR10-HTR10:RFP in the vegetative cell and sperm cells were respectively reduced by 78 (*n* = 1,885), 47 (*n* = 2,211), and 60% (*n* = 3,889) on the *akrp-3/*+ background (Supplementary Figures S3f,g), indicative of *akrp*-3 pollen effect on vegetative and sperm cell differentiation. Rarely, some of the *akrp*-3 bi-cellular pollen or those containing cytoplasmic inclusions expressed both vegetative and sperm cell markers (Supplementary Figures S3f,g). When germinated *in vitro*, only a small population of bicellular PTs was observed, and its growth appeared retarded, with no or rare expression of cell fate markers (Supplementary Figures S3f,g). Collectively, these results suggest that the *akrp-3* loss of function mutant failed to correctly differentiate the male and female gametic cell fates. However, selfed *akrp*-3/+ heterozygous plants did not show significant segregation distortion, whereas reciprocal test crosses showed a decreased transmission only through the female (78% TE), but not through the male gametophyte (97% TE) (Supplementary Figures S4a,b).

### Arabidopsis Ankyrin Repeat Protein in Ovules Is Required for Pollen Tube Attraction

**FIGURE 2:**
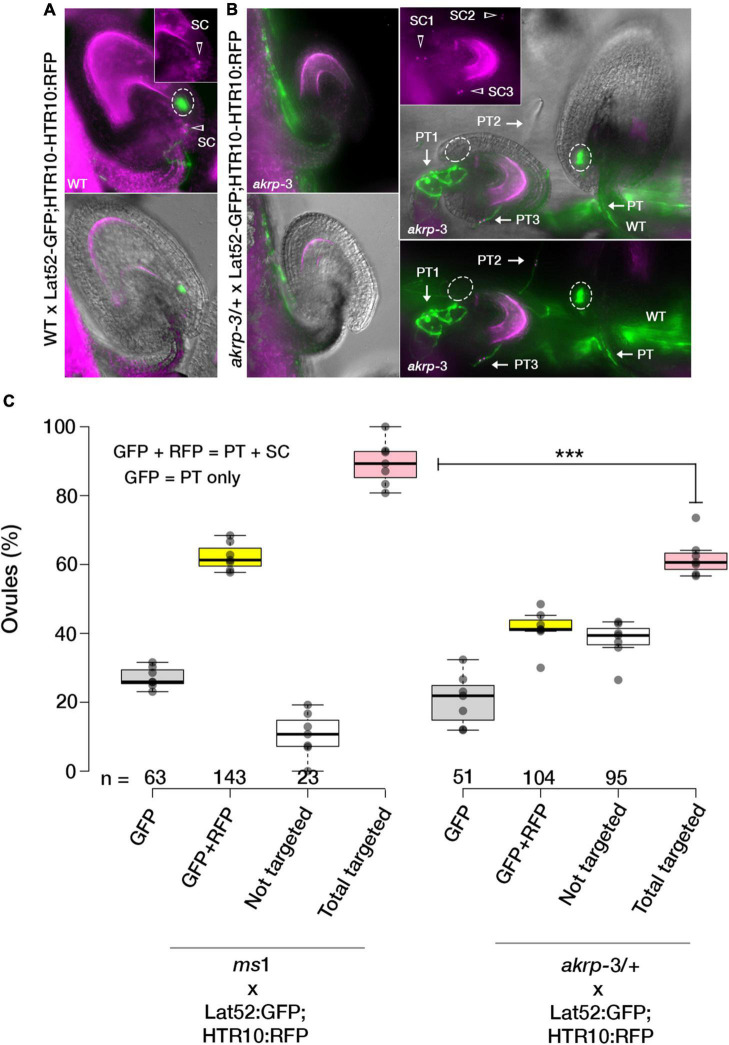
The AKRP function in ovules is critical for pollen tube attraction. Double fertilization events in WT and *akrp*-3/+ pistils were observed after pollination with a double marker for sperm cells (pHTR10-HTR10:RFP) and vegetative cell (pLat52-GFP) 24 h after pollination. **(A)** Normal ovule targeting and reception are scored as a uniform GFP halo (ellipses) at the micropylar and presence of sperm cells (arrow head). **(B)** In *akrp*-3/+ pistil, a mixture of normal targeted and non-targeted ovules or with attraction/reception defects including up to three pollen tubes (arrows) without entry or display of GFP halo were observed. **(C)** Box plot quantification of attraction defects in respective crosses. Center lines represent the medians, and dots outside the quartile are outlier pistils, *n* = 175 and 166 ovules respectively. Statistics were conducted by two-sided non-parametric multiple comparisons with Dunnett contrast and logit approximation, ***p < 0.0005. PT, pollen tube; SC, sperm cells. Scale bar = 50 μm.

In *akrp*-3/+ siliques, a high percentage of seed gaps appeared, indicating a likely fertilization defect. To establish whether untargeted ovules resulting in collapse ovules are those with *akrp-3* loss of function, we conducted a live cell imaging to visualize a correct PT reception (PT arrest, burst, and sperm cell release in the receptive synergid cell) in a*krp-3/*+ pistils. Flowers were emasculated and pollinated with a homozygous double marker for sperm cells (pHTR10-HTR10:RFP) and vegetative cell (pLat52-GFP) (Figures 2A,B). Twenty-four hours after pollination, ovules in pollinated pistils were exposed by dissecting the carpel walls, and confocal live-cell imaging was performed to visualize the position of GFP-labeled pollen tubes and HTR10-RFP-labeled sperm cells in wild-type and *akrp-3* ovules. On average, 43.1% (*n* = 250) of the ovules in *akrp*-3/+ pistil did not attract any PTs and, thus, were not fertilized (Figures 2B,C). In some infrequent events, mutant *akrp*-3 ovules attracted multiple PTs containing sperm cells, a phenomenon termed polytubey (Beale et al., 2012; Maruyama et al., 2013). However, none of the PTs appeared to enter the mutant *akr* ovules and burst to release sperm cells (Figure 2C). An independent analysis using a blue dot assay with Lat52-GUS pollen unveiled a reduced PT targeting in the *akrp*-3/+ pistil, where nearly 46% (*n* = 264) of the ovules were not targeted by the Lat52-GUS-expressing PT (Supplementary Figure S4c). These data support the live-cell imaging observations and, together, imply that *akrp*-3 ovules are incompetent in PT attraction for fertilization.

### A Fraction of Targeted *akrp* Ovules Exhibits a Range of Pollen Tube Reception Defects and Fails to Develop an Embryo

**FIGURE 3:**
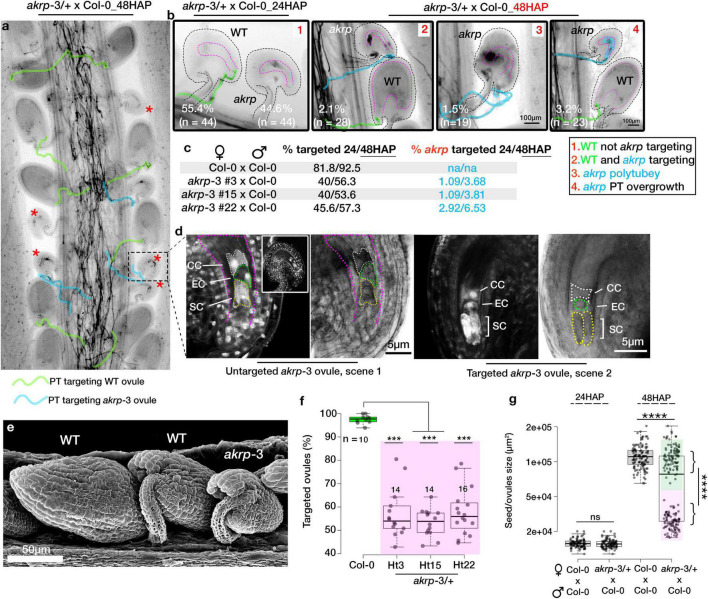
Fraction of targeted *akrp* ovules fails to develop an embryo. *In vivo* pollen tube attraction between Col-0 × Col-0 and *akrp*-3/+ × Col-0 was assayed 24 and 48 h after pollination (HAP). **(a)** Representative pistil section from *akrp*-3/+ × Col-0 stained for callose showing wild-type (unmarked) and mutant ovules (red asterisks) and pollen tube traces (PT) targeting respective ovules. **(b)** Four phenotypic scenarios at 24 and 48 HAP were observed in *akrp*-3/+ × Col-0 pistil but not in wild-type pistil. n represents the number of pistils analyzed. **(c)** Quantification of total targeted ovules and a fraction of *akrp*-3 targeted ovules 24 and 48 HAP using both types of heterozygous *akrp*-3 with single or back-to-back insertion (Figure 1C). An increase in *akrp*-3 targeting can be seen 48 HAP. **(d)** Despite *akrp*-3 ovule targeting, the majority of the *akrp-3* ovules do not produce an embryo, and the female gametophyte remains unfertilized. **(e)** Scanning electron microscopy images using secondary electron detector of *akrp*-3/+ pistil pollinated with Col-0 pollen 48 HAP demonstrated clear presence of underdeveloped *akrp-3* seeds despite potential pollen tube targeting. **(f)** Variability in the frequency of ovule targeting as seen in individual pistils 48 HAP from respective genotypes. **(g)** Estimation of seed/ovule sizes 48 HAP clearly highlighting lack of fertilization and development of the majority of *akrp*-3 ovules. Statistics were conducted by two-sided non-parametric multiple comparisons with Dunnett contrast and logit approximation or by Student’s *t*-test, ***/****p < 0.0005/0.0001. ns, not significant. PT, pollen tube; SC, synergid cell; EC, egg cell; CC, central cell.

The tendency of *akrp*-3 ovules we observed in live cell imaging not to attract PTs or attract multiple PTs without PT burst prompted us to investigate the frequency of PT attraction by *akrp*-3 ovules. We additionally questioned whether *akrp*-3 attracted PT experience PT reception defects and/or fertilization blockage. Since pollen tube reception and fertilization improve with longer hours after pollination (HAP) (Grossniklaus, 2017; Johnson et al., 2019; Nagahara et al., 2021), we manually crossed Col-0 × Col-0 and compared with *akrp*-3/+ × Col-0 24-to-48 HAP. Callose staining of in-pistil PTs revealed a similar *akrp*-3 untargeted event 24 HAP to that observed by live-cell imaging and the blue dot assay as the predominant phenotype (Figure 3a). However, a careful look unveiled that the tendency of *akrp*-3 PT attraction, polytubey attraction, as well as PT reception defects (PT overgrowth), increased at the 48 HAP time point (Figures 3b,c). We noticed, on average, that about 2% (*n* = 28) of *akrp*-3 ovules are targeted and arrested PT at micropylar entry, although without clear pollen tube burst (Figure 3b, scene 2). Another 1.5% (*n* = 19) also showed polytubey without clear PT entry, and majority of *akrp*-3-targeted ovules (3.2%, *n* = 23) showed a PT overgrowth, indicating a PT reception defect (Figure 3b, scenes 3 and 4 respectively). Nevertheless, this suggests that if given time, some of the *akrp*-3 ovules (up to 6.5%, *n* = 70 pistils) can eventually at least attract PTs or be targeted by a PT (Figure 3c). But, do these *akrp*-3-targeted ovules undergo fertilization? Whether targeted by a PT or not, mutant *akrp*-3 ovules lag behind in development, as they are clearly distinguishable by their small size at the 48 HAP time point (Figure 3a). Dissection of these retarded ovules revealed no presence of initiated embryos in *akrp*-3 ovules and the female gametic cells remained clearly visible and unfertilized 48 HAP (Figure 3d). At this time point, the wild-type targeted ovules are at the minimum 32-cell embryo stage (Figure 3d). We, therefore, conclude that although a fraction of *akrp*-3 ovules is successfully targeted, they likely exhibit later PT reception incompetence, as seen in PT overgrowth or the inability to induce PT burst, and, therefore, do not undergo fertilization. Forty-eight hours after pollination, on average, 55.8% (*n* = 44 pistils) of the ovules (corresponding to wild-type ovules) were correctly targeted in *akrp*-3/+ pistils pollinated by Col-0 pollen compared to 92.5% (*n* = 14 pistils) targeted ovules in Col-0 pistils also pollinated by Col-0 (Figure 3e). To support the observation that the majority of *akrp*-3 ovules do not proceed with fertilization and embryo development, we measured seed area 24 and 48 HAP in Col-0 and *akrp*-3/+ pistils pollinated with Col-0 pollen. Our analysis identified two distinguishable populations at 48 HAP in the *akrp*-3/+ pistils but not in the Col-0 pistils, supporting lack of fertilization and embryogenesis of Col-0 targeted mutant *akrp*-3 ovules (Figure 3f and Supplementary Figure S4d).

### Arabidopsis Ankyrin Repeat Protein Post-fertilization Is Essential for Embryo Progression and Chlorophyll Synthesis

**FIGURE 4:**
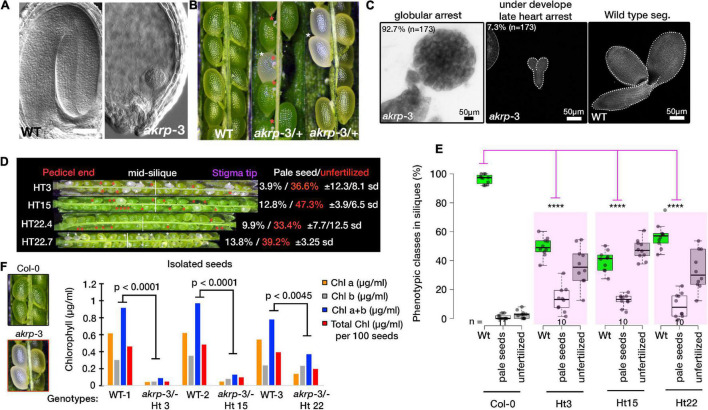
Homozygous *akrp* arrests at globular embryo and is depleted of chlorophyll synthesis. **(A,B)** Fully developed seedlings of wild-type and globular arrested embryo of *akrp*-3 from cleared seeds of WT (green) and *akrp*-3 (pale, labeled with white asterisks, and red asterisks mark unfertilized *akrp*-3 ovules). Some siliques showed a more unfertilized phenotype over embryo arrest as quantified in panel **(D)**. **(C)** Although globular embryo arrest is a dominant *akrp*-3 embryo phenotype, some *akrp*-3 embryos halt their development in the later heart stage. **(D)** Phenotypic penetrance among *akrp*-3/+ hemizygous individuals from a common parent showing variation in the appearance of unfertilized ovules (red asterisks) and a pale embryo lethal phenotype (white asterisks). *N* = 15 siliques per genotype. **(E)** Measured frequency and variation of the *akrp*-3/+ seeds phenotype at silique maturity. **(F)** Chlorophyll measurements from isolated seeds of the respective genotypes. Data represent an average of pooled triplicates. Statistical analysis was performed by one-tail Student’s *t*-test assuming unequal variance. ****p < 0.0001.

Self-pollinated *akrp*-3/+ generates, on average, 10% of homozygous *akrp* pale mutant seeds on top of the 44.2% average aborted ovule phenotype (Figures 4A,B). This suggests that mutant *akrp*-3 gametes from both gametophytes can get together and initiate an embryo in the aforementioned frequency. Isolation and dissection of the mutant pale seeds revealed that approximately 93% (*n* = 173) *akrp*-3 embryos are arrested at the globular stage, with the remaining 7% proceeding to late heart stage (Figure 4C), while the wild type segregant and Col-0 control had completed cotyledon development at similar time point (Figure 4C). Because of the genotyping variation we observed (Figure 1C), we investigated if this has an association with the *akrp*-3/+ silique phenotype. We dissected siliques from HT3, HT15, and HT22 (from Figure 1C) and observed the variable penetrance of pale seeds and unfertilized ovule phenotypes that were independent of genotypic origin (Figures 4D,E). Since mutant *akrp*-3 ovules are phenotypically without pigments (Figure 4F), we isolated mutant seeds and measured chlorophylls a and b, and total chlorophyll levels. All chlorophylls a and b levels as well as total chlorophyll were significantly depleted in the mutant *akrp*-3 seeds, suggesting lack of chlorophyll synthesis in the *akrp*-3 mutant seeds.

### Arabidopsis Ankyrin Repeat Protein Localized to Proplastid in Gametophyte and in Isolated Chloroplast

**FIGURE 5:**
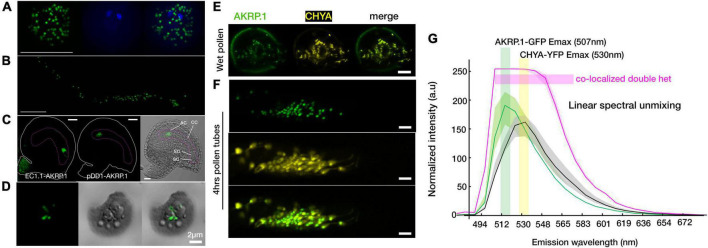
Arabidopsis ankyrin repeat protein localized to the plastid in gametophyte and in an isolated chloroplast. **(A,B)** Punctate AKRP.1::GFP localization in pollen and *in vitro* pollen tubes expressed with the pollen-specific Lat52 promoter. **(C)** Under the egg cell promoter EC.1.1 (left panel) and an antipodal specific promoter DD1 (ubiquitin-conjugating enzyme 31, AT1G36340) (middle and right panels), AKRP.1::GFP punctate localization were observed specifically in the egg cell and antipodal cells of the wild-type embryo sac. **(D)** In isolated chloroplast, AKRP.1::GFP localized mainly in the stroma of the central chloroplast. **(E)** Co-expression of pLat52- AKRP::GFP with a plastid specific marker, pBINU-CHYA(K), in the pollen and **(F)** pollen tubes revealed strong co-localization and confirmed AKRP plastid localization in the gametophyte. **(G)** Representative linear unmixing spectra plot from double hemizygous pollen tubes for both constructs, *N* = 55 pollen tubes, ×32 ROI each. Quantification was performed with the ImageJ plug-in using fixed-size ROI. This panel is also supported by Supplementary Figure S6. Scale bar = 10 μm.

A previous study colocalized 35S-spAKRP:GFP (first 50 amino acids from AKRP containing N-terminal transit peptide) with chlorophyll autofluorescence in transiently transformed *Nicotiana benthamiana* guard cells but not the full length 35S-AKRP:GFP as proven deleterious to the chloroplast (Garcion et al., 2006). More recently, both EMB506 (STT1) and AKRP (STT2), under the control of their endogenous promoters, were reported to localize in a punctate pattern in the chloroplasts of an isolated protoplast (Ouyang et al., 2020). However, because of weakness of the *AKRP* promoter, we could not reliably detect any visible GFP signal in stable pAKRP-AKRP:GFP transformants. Nevertheless, the expression of AKRP under pLat52 (pollen vegetative cell-specific), egg cell specific EC1.1, or pDD1 (antipode-specific, AT1G36340, Steffen et al., 2007) promoters clearly localized the AKRP-GFP fusion protein into likely proplastids or differentiated elaioplasts in mature pollen, pollen tubes, the egg cell, and antipodal cells of the female gametophyte (Figures 5A–C). Only egg cell localization appeared less punctate and more cytoplasmically distributed (Figure 5C). We also isolated chloroplast from transiently transformed *Nicotiana benthamiana* leaves expressing a full-length AKRP-GFP and detected an AKRP localization in the chloroplast stroma (Figure 5D). To confirm AKRP-GFP plastid localization in pollen, we co-transformed pLat52-AKRP:GFP plants with a UBIQUITIN 10 promoter-driven pBINU-CHYA vector expressing 85 amino acids of the chloroplast NADPH-dependent thioredoxin reductase C (NTRC) destined to the chloroplast stroma fused to an aequorin calcium sensor and a YFP fluorophore (Mehlmer et al., 2012). Both fluorophores strongly colocalized in proplastids or differentiated elaioplasts of the pollen and pollen tubes, as supported by the GFP-YFP spectral overlap and online emission fingerprint (Figures 5E–G). These results confirmed AKRP-NTRC plastid co-localization (Figure 5G). Similar results from mature pollen were obtained by independent channel excitation (Supplementary Figure S6). Collectively, these data provide evidence that AKRP is likely to function in plastid differentiation and in chloroplast.

### Cell-Type-Specific Complementation of *akrp-3* Revealed Timing of Arabidopsis Ankyrin Repeat Protein Function

To confirm the *akrp-3* unique gametophytic phenotype that was not observed in other alleles, we performed-complementation using a native AKRP promoter and cell type-specific promoters in an attempt to identify the developmental timing at which AKRP functions.

**FIGURE 6:**
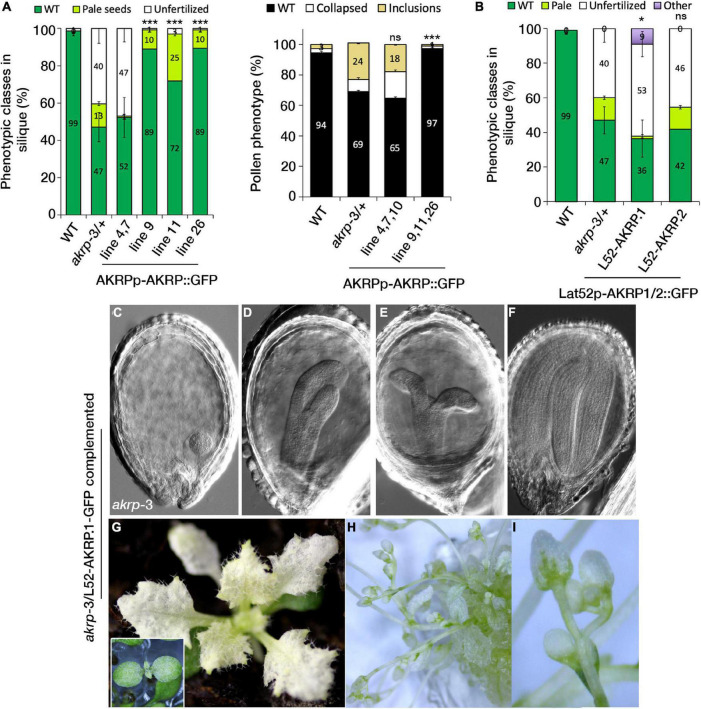
Cell type-specific expression of *AKRP* longer isoform partially rescues embryo developmental perturbation and reveals the timing of AKRP function. **(A)** Native promoter-driven complementation resulted in two groups of complemented plants: those with reduced embryo phenotype but persistent gametophytic defects (lines 4, 7, and 10) and those with fully restored pollen defects (right panel) but with remnant pale seed embryo defects (lines 9, 11, and 26). **(B–I)** Complementation of *akrp*-3/+ with the pLat52-AKRP.1::GFP longer isoform partially complemented the *akrp-3* embryo defect (“other” category), producing rescued embryos beyond the globular arrested embryo of *akrp*-3. Some *akrp*-3 homozygote seeds were able to germinate and flower *in vitro*. Statistical tests were performed by Student’s *t*-test assuming unequal variance, *p < 0.05, ***p < 0.001; ns, not significant. Scale bar = 50 μm.

We fused the complete AKRP genomic fragment, including the 997-bp intergenic fragment, as a putative promoter region to N-terminal eGFP and transformed mutant *akrp*-3/+ plants. Intriguingly, the introduction of AKRP-GFP either complemented both male and female *akrp*-3 gametophytic defects but not fully the embryo defect (lines 9, 11, and 26), or resulted in increased frequency of gametophytic defects and reduced embryo defects (lines 4, 7, and 10) (Figure 6A). In line 11, the complete rescue of both gametophytic *akrp* defects resulted in an increased average of pale *akrp*-3 homozygous mutant embryos, suggesting a possible uncoupling of gametophytic and embryogenic AKRP functions (Figure 6A). However, such individuals were underrepresented among the complemented lines.

Since AKRP encodes two splice isoforms, AKRP.1 and AKRP.2 (Figures 1A,B and Supplementary Figure S1), we tested both isoforms for their ability to complement the *akrp*-3/+ phenotype in a cell-specific manner. Using pLat52-AKRP.1/2::GFP constructs, we identified that only the longer isoform (AKRP.1) partially rescued the embryo-lethal phenotype but not the pollen phenotype, and that the gametophytes remained predominantly sterile (Figure 6B). This suggests that the time window for AKRP activity is much earlier during pollen maturation (as shown by the AKRP promoter activity) compared to the late onset of the Lat52 promoter activity (bicellular pollen), and that the shorter isoform is unlikely to function during the embryo development. Most of the Lat52-AKRP.1 rescued embryos were able to develop much further, forming complete cotyledons, compared to the original globular arrested *akrp-3* embryos (Figures 6C–F). The rescued embryos still exhibited cotyledons malformations and lacked the normal levels of chlorophyll; therefore, they remained white to pale green even during the vegetative phase in soil (Figure 6G). To establish how far the rescued embryos could develop, *akrp*-3/*akrp*-3 germinated seedlings were transferred into glass jars containing half MS medium and grown *in vitro* under 16-h/8-h light/dark cycle. Some of the complemented lines were able to produce flowers, although with long delays and high sterility (with only scarce seed set) compared to the control wild- type plants (Figures 6H,I).

To get a broader perspective of AKRP function, we complemented the *akrp-3/*+ plants with a CaMV 35S promoter-driven AKRP.1:GFP. Although the seeds remained pale, we again observed a partial 35S-AKRP.1 complementation of the pale seedlings, which later recovered the cotyledon chlorotic phenotype and produced viable flowering plants (Supplementary Figures S5a–e). While some of the lines retained a patchy white-green pattern on leaves, the others fully recovered and produced green organelles (Supplementary Figures S5c,d). A similar patchy appearance was observed when the 35S-AKRP:GFP construct was introduced into WT Col-0, suggesting that the observed phenotype was likely due to the nature of 35S overexpression-induced silencing.

To assess AKRP function in the female gametophyte, two promoters driving the expression of the longer *AKRP.1* isoform were used: an antipode-specific DD1 (AT3G36340) as a potential negative control for embryo sac cell-specific complementation and an egg cell-specific EC1.1 (AT1G76750) promoter. Intriguingly, the pEC1.1-driven AKRP complementation was not sufficient to rescue the embryo-lethal phenotype or the ovule-pollen tube attraction defect of *akrp*-3 (Supplementary Figure S5f). However, the pDD1-driven complementation construct was able to partially rescue the embryo developmental defects. Several lines were recovered with fully developed embryo; however, some had pale seeds that resembled the pLat52-AKRP:GFP-complemented lines (Supplementary Figure S5g). This suggests that the spatial-temporal DD1 promoter activity is sufficient to drive the AKRP embryo function.

## Discussion

In this study, we have identified and functionally associated AKRP with a role in gametophyte competence to undergo fertilization. Previous studies highlighted the role of AKRP in embryonic development, but the alleles studied exhibited no gametophytic defects (Garcion et al., 2006; Meinke et al., 2008). The AKRP falls under the category of embryo-defective genes (EMB) where the fusion of two mutant gametes results in a lethal embryonic development. We show for the first time that AKRP functions earlier not only during embryonic development but also at the gametophytic level (Figures 1–3). In Arabidopsis, it is not yet clear how many EMB genes also have a role in the gametophyte, but 27% of the EMB genes exhibit embryo arrest in the globular stage, same stage as the *akrp*-3 mutant, whereas the others arrest earlier or later pass the globular stage, implying a differential timing of EMB gene function (Muralla et al., 2011; Meinke, 2019).

### Putative Timing of Arabidopsis Ankyrin Repeat Protein Function in Gametophyte and Embryonic Development

An emerging role of plastids during pollen maturation, as assessed by transmission electron microscope, suggests an essential role in carbohydrate storage that serves to provide energy and monomers for the construction of a PT wall. Moreover, in *Lolium perenne*, plastids store brassinosteroids during pollen maturation and later promote PT growth post pollination in stigmatic papillae and in transmitting tract tissues (Taylor et al., 1993). Therefore, the timing of genes associated with plastid biogenesis is crucial in the gametophyte and post fertilization.

The rescue of the *akrp*-3 embryo phenotype but not the pollen phenotype by the Lat52-AKRP:GFP construct is spatially intriguing. We suspect a possible inheritance of pollen-expressed AKRP: GFP post-fertilization. This possibility of sporophyte-to-gametophyte carryover inheritance (meiocytes to microspores in the male gametophyte), which masks the gametophytic phenotype, or gametophytic-to-diploid zygote carryover inheritance (ovule or pollen tube-to-zygote during double fertilization), which rescues early embryo defects, has been previously reported for several genes (Muralla et al., 2011; Nodine and Bartel, 2012; Meinke, 2019). The Lat52 promoter is known to be active only in pollen (Ursin et al., 1989; Twell et al., 1990); however, its activity was also reported in roots, as verified by protein gel blot analysis (Van Damme et al., 2006). Therefore, it is possible that the ectopic activity of the Lat52 promoter post-fertilization might be an alternative explanation over a pollen-originated AKRP transcript inheritance for the sufficient rescue of the *akrp-3* embryo phenotype. Partial complementation of the *akrp* embryo phenotype has also been previously reported by Garcion et al. (2006) using an embryo-specific promoter AB13, implying that the timing of AKRP expression is crucial for its function. Similar explanation could be hypothesized for the lack of *akrp*-3 pollen phenotype complementation by the pLat52-driven construct. The LAT52 promoter is active in vegetative cell from the bicellular stage (Brownfield et al., 2009a,b), whereas the AKRP promoter was most active in the earlier microspore stage and the tapetum (Supplementary Figures S2c–f), and the first defects in *akrp-3/*+ pollen morphology were already visible in the bicellular pollen stage. As such, Lat52-driven AKRP expression is far late to rescue the *akrp*-3 pollen phenotype, as supported by the lack of *akrp*-3 pollen complementation we observed (Figure 6B).

Interestingly, in the embryo sac, the antipodal cell-specific expression of AKRP.1 by the DD1 promoter, but not the egg cell-specific expression by EC1.1 promoter, partially complemented the *akrp*-3 globular arrest embryo phenotype (Figures 4, 6). The antipodal-driven complementation of *akrp*-3 embryo arrest was unexpected because of the fact that antipodal cells degenerate post fertilization. We suspect that, perhaps, the window of expression by the DD1 promoter was sufficient to accumulate AKRP.1 transcripts to allow for AKRP.1 function post fertilization. On the other hand, the lack of *akrp*-3 complementation through egg cell-specific expression by the EC1.1 promoter can be explained by the fact that the EC1.1 promoter itself did not appear activated in *akrp*-3 ovules; therefore, the EC1.1-AKRP.1:GFP construct was not active in the *akrp*-3 egg cell (Supplementary Figure S3g). It will be informative in the future to express the AKRP.1, specifically in the egg cell, using a different promoter.

We also observed *akrp*-3 embryo complementation by 35S-AKRP: GFP. The construct seemed to improve pollen fitness in the rescued homozygous lines (Supplementary Figure S4). However, some of the rescued lines lacked pigmentation and developed deformation on leaves. The chlorotic patchiness in leaves might be the result of overexpression-induced silencing of AKRP in developing embryos. The AKRP-coding sequence contains a + 31-1,110-bp nonsense-mediated decay target sequence (Thierry-Mieg and Thierry-Mieg, 2006); therefore, its strong expression by the 35S promoter could likely induce silencing. A similar observation was reported by Zhang et al. (1992, 1994) from multiple transgenic lines with chlorotic phenotype after transformation with both sense and antisense AKRP constructs. A similar construct was also used to complement the *emb2036*-1 and generated homozygotes lines with a similar outcome (Garcion et al., 2006). In summary, our complementation analyses hinted that if the promoter used has a broader pattern of expression in either of the gametophytes (female proDD1 or male proLat52), it was sufficient to rescue the lethal embryo arrest phenotype. This is potentially substantiated through the inheritance of parental-originated macromolecules (transcripts or proteins) during double fertilization. The next step will be to structurally dissect isolated gametophytic plastids (predominantly from the vegetative cell of pollen and developing ovules) from *akrp*-3 mutant to evaluate their structures relative to those from the wild type. A similar analysis on developing embryos revealed a striking difference and assigned a role of AKRP in plastid differentiation (Garcion et al., 2006).

### *EMB-*Defective Allele Non-canonical Penetrance

The two other mutant alleles of AKRP, *emb2036-1* and *emb2036-2*, are phenocopies, both showing 25% homozygous embryo arrest at the globular stage (Tzafrir et al., 2004; Meinke et al., 2008). Here, we have screened three alleles of *AKRP*, *emb2036-1*, *akrp-3*, and *akrp-4*. *emb2036-1*, with T-DNA inserted at the 5UTR showing no gametophytic defects, whereas *akrp-3* loss of function, with T-DNA inserted at the beginning of exon 1 exhibiting various pollen, ovule, and embryo defects (Figures 1–3). We suspect that these allelic variations might also be explained by T-DNA positional effect. The *AKRP* encodes two isoforms that we verified to be expressed in pollen, ovules, and seeds with the longer isoform up to three times more abundance (Figure 1). In pollen, only the longer isoform can complement the pollen defect, and in *akrp*-3 knockout, both isoforms are disrupted (Figure 1). It is unclear to us whether the *emb2036-1* and *emb2036-2* alleles that exhibit only the embryo phenotype result in the disruption of both isoforms. Because of the close proximity of AKRP with the neighboring AT5G66060 gene sharing the putative promoter region, our screening confirmed that none of the AT5G66060-associated T-DNA insertions exhibit any aberrant phenotypes. Together with the complementation of *akrp*-3 by the AKRP.1-GFP native construct, this confirms that the gametophytic phenotype we observed is linked to *akrp-3* loss of function.

Despite the substantial amount of aberrant pollen with altered cell fate and significant lack of pollen tube attraction leading to an ovule abortion in *akrp*-3, there were no transmission defects through the male and only a reduced transmission (78%) through the female. This reduction does not correspond to the penetrance of the *akrp*-3 gametophytic phenotype (Figures 1–3 and Supplementary Figure S4). A similar example of *akrp*-3 non-characteristic pattern of allele inheritance was observed in another plastidial isoform of NAD-MDH that produced NAD + required to generate ATP by glycolysis. Knockout plNAD-MDH is embryo-lethal and exhibits impaired pollen tube growth *in vitro*, which can be rescued by exogenous application of NADH-GOGAT substrates (Selinski and Scheibe, 2014). Just like *akrp*-3 pollen tubes, *plnad-mdh in vivo*-grown pollen tubes are functional and able to fully compete with the wild-type pollen tubes for fertilization (Selinski and Scheibe, 2014). This explains the normal transmission of the *plnad-mdh* mutant allele through the male despite the pollen and pollen tube phenotype. These results suggest that *in vivo*, female reproductive tissues play a key role in supporting semi-functional pollen tubes compromised by the knockout of some essential loci.

Allelic variation in *emb* mutants is also not uncommon. A T-DNA insertion in the GEX1 gene produces a truncated fragment sufficient to rescue a *gex1* gametophyte defect but not an embryo defect (Alandete-Saez et al., 2011). Similarly, a truncated fragment of ZAR1 receptor kinase results in dominant-negative effect on embryo development, a phenotype not visible in other *zar1* null alleles (Yu et al., 2016). A more similar example of *akrp*-3 allelic variation is disruption of geranylgeranyl diphosphate synthase required for isoprenoid biosynthesis that also encodes two isoforms, a short isoform and a longer isoform (Ruiz-Sola et al., 2016). A T-DNA disrupting only the longer isoform which is targeted to plastids shows a defect in seedling pigmentation, whereas disruption of the shorter isoform, exhibits embryo lethal phenotype (Ruiz-Sola et al., 2016).

Extensive screening of *emb*-defective genes by integrated multi-omics analysis has produced a consistent phenotypic profile that is transcription-linked to elaborate the behavior of *emb*-defective genes, including allelic variation, and their potential unusual inheritance (Muralla et al., 2011). Among *EMB* loci categories, those with functional male gametophyte but with pre-globular embryo arrest, 85% of the *EMB* genes in this category are maintained as heterozygous, and their transcripts are detected pre-meiosis in microsporocytes (pollen mother cell) and then in early microspores, suggesting a transcript inheritance from the microsporocytes to the microspores (Muralla et al., 2011; Meinke, 2019). By anthesis, their transcripts disappear. This is consistent with the masking of their gametophytic phenotype through a “maternal rescue,” and they, instead, exhibit an onset of early embryo arrest phenotype. In other *EMB* genes with moderate-to-severe male gametophyte defect, their transcription tends to be throughout the pollen development with later onset of embryo arrest phenotype. *De novo* post-meiotic expression (over pre-meiotic transcript inheritance) is, therefore, likely essential for this category of *EMB* genes to allow sufficient levels for gene function throughout pollen ontogenesis. The profile of *akrp*-3 perfectly fits to the second category, exhibiting mild pollen phenotype yet functional to allow for normal transmission of the mutant *akrp*-3 allele. Transcripts of AKRP are detectable in microsporocytes and drastically decreased during pollen maturation, and the *akrp*-3 exhibits a pre-globular embryo arrest (Figures 1, 4). The mild pollen phenotype also suggests that either *de novo* AKRP post-meiotic transcription is necessary for full AKRP function, or the *akrp*-3 allele that disrupts both isoforms of *AKRP* is a true null allele over the previously reported *emb2036*-1 and *emb2036*-2 alleles that did not exhibit the gametophytic phenotype. To summarize, RNA and/or protein storage in the gametophyte is emerging as a substantial element of sporophyte-gametophyte reinforced fitness in flowering plants to support the isolated gametophyte function for successful fertilization and sustain an early embryo development pre-zygotic gene activation. The complexity of an EMBRYO-DEFECTIVE gene (EMB) allelic variations and the non-canonical allelic inheritance patterns have recently been reviewed by the group of David W. Meinke, who initiated and characterized over 1,000 *emb* mutants more than 40 years ago. The resources presented in his review (Meinke, 2019) and the curated database, SeedGenes^2^, exclusively documenting *emb* mutants, with gametophytic or embryo developmental role, are extremely valuable and are a must-explore data source.

### Mechanistic Insight Into the Function of Arabidopsis Ankyrin Repeat Protein

Per cell, a chloroplast contains over 3,000 proteins; the majority of which are nuclear-encoded. These are synthesized in the cytosol with an N-terminal transit peptide and are subsequently translocated into chloroplasts (Jarvis and Loìpez-Juez, 2013; Lee et al., 2017). Chloroplasts constitute of multi-suborganellar membranes, outer envelope, inner envelope, and thylakoid membranes, and thus create three separate compartments, the intermembrane space, stroma, and the lumen (Ouyang et al., 2020). Therefore, nuclear-imported proteins need to be further resolved into subcellular organelles. The initial import into the stroma is done by the Toc/Tic import complex located in the outer/inner membrane of the chloroplast *via* N-terminal transit peptide recognition (Jarvis and Loìpez-Juez, 2013; Chen et al., 2018). To further sort nuclear-imported proteins, thylakoid membrane-destined proteins follow the chloroplast signal recognition particle pathway (RP), whereas thylakoid lumen proteins are sorted using an additional targeting signal at their N-terminal and enter either the chloroplast secretory (cpSec) or the chloroplast twin-arginine translocation (cpTat) pathway (Schuenemann et al., 1998; Yuan et al., 1994; Settles et al., 1997). Recently, AKRP (STT2) and EMB506 (STT1) were shown to be integral in the formation of liquid-liquid phase-separated droplets as a mechanistic novel mechanism of intra-chloroplast cargo sorting *via* the cpTat pathway to transport thylakoid membrane proteins (Ouyang et al., 2020). STT1-STT2 interacts to create a heterodimer and *via* N-terminal intrinsically disordered regions of the STT complex induces liquid-liquid phase separation (Ouyang et al., 2020). The RNAi silencing of either STT1 or STT2 led to a defective thylakoid membrane biogenesis and plastid morphogenesis, resulting in growth retardation, chlorotic leaves, and disrupted chlorophyll levels.

## Conclusion

We report for the first time that the role of AKRP in intra-chloroplastic cargo protein sorting *via* a liquid-liquid phase translocon driven separation (Ouyang et al., 2020), plays a crucial role not only in pigmentation and embryonic development, but also in gametophytic-fertilization competence likely *via* plastidial morphogenetic function. Our data also suggest that this AKRP role is extended throughout plant development in sorting chloroplast import proteins from crowded stroma to thylakoid membranes, as partially rescued *akrp*-3 adult plants remain chlorotic with morphological distortion throughout plant development.

## Materials and Methods

### Plant Material and Growth Conditions

Arabidopsis (*Arabidopsis thaliana* L. Heynh.) plants were grown at 22 and 60% humidity in Conviron PGC Flex growth chambers under 16-h light/8-h dark conditions. Seeds of Col-0 wild type (WT) and T-DNA lines of EMB2036/AKRP (At5g66055), and At5g66060 plants were obtained from The European *Arabidopsis* Stock Centre. Segregating T-DNA line Gabi-Kat GK_876D05, located in the first exon (in reverse orientation upstream of nucleotide 614) of AKRP was the main experimental material used in this study and herein referred to as *emb2036-3*. The second studied T-DNA line was SAIL_98_F02 (*emb2036-4*, located in 5′UTR). For At5g66060, located on chromosome 5 downstream of AKRP, three T-DNA insertion lines were grown (SALK_098611C, SAIL_595_H12, and SAIL_1262_D08). The T-DNA lines were genotyped using either Gabi-Kat o8474 or o8760, SALK LBb1.3, or SAIL LB2 primers in combination with gene-specific primers. Primer sequences are listed in Supplementary Table S1.

### Phenotypic Screening

The T-DNA insertion lines were screened for defects in pollen and embryo development phenotypes. Mature pollen samples were screened in brightfield and UV on a Nikon TE-2000 microscope. Siliques in the cotyledon stage of embryo development were dissected and screened for the presence of pale white/transparent mutant seeds. The phenotypic screening was conducted on two subsequent generations.

### Transmission Analysis

The *emb2036-3* heterozygous plants were back-crossed into Col-0 wild-type background to determine female transmission and crossed into male-sterile *ms1-/-* (Yang et al., 2007) to study male transmission. The progeny of these crosses was genotyped by PCR for presence of T-DNA insertion, and transmission efficiency (number of seedlings containing *emb2036-3* insertion/number of wild-type seedlings) was calculated. Siliques from the reciprocal crosses were screened for the presence of gametophytic or embryo phenotypes that could be caused by a single mutant parent.

### Blue Dot Assay

Young buds of WT Col-0 and *emb2036-3/*+ plants before dehiscence of stamens were emasculated and let to mature for 2-3 days until stigma papillae developed. The pistils were then pollinated with pollen containing the pLat52-GUS construct and collected 24 h after pollination. Fertilized pistils were dissected under a binocular dissection microscope (Leica, Germany), and the stripes of fertilized ovules attached to the septum were transferred to a GUS staining solution (view section Histochemical GUS staining). After 2 h of staining, fertilized ovules were observed under the Nikon TE-2000 microscope for the presence of a blue dot following pollen tube micropylar entry and burst.

### Pollen Viability Stain and Aniline Blue Staining

Alexander staining was performed according to the protocol by Schoft et al. (2011). Stamens of WT and *akrp-3/*+ plants were stained in a droplet of the solution. For aniline blue staining, pistils of 24-h self-pollinated WT Col-0 and *akrp-3/*+ plants or cross-pollinated *ms1* plants with WT or *akrp-3/*+ pollen (24-h growth *in vivo*) were stained for callose according to the protocol by Mori et al., 2006.

### Pollen Tube Cultivation

*In vitro* pollen tube growth was performed according to the protocol by Boavida and McCormick (2007). After 8 h of *in vitro* growth, pollen germination rate and pollen tube length were measured using the NIS Elements software. For semi-*in vivo* pollen tube growth assay, sterile *ms1/-* pistils were pollinated with WT Col-0 or *akrp-3/*+ pollen, and pistils were collected 1 h after pollination. The pistils were excised at the stigma shoulder with a needle and transferred to the growth medium (Palanivelu and Preuss, 2006) on a small Petri dish. The pistils were tilted to enable the pollen tubes to emerge on the surface of the solid medium and cultivated in a humidified growth chamber at 22°C for 24 h. Pollen tube length was measured using the NIS Elements software from the point where they emerged from the cut pistil.

### Chloroplast Isolation and Chlorophyll Measurements

Six leaves of old *Nicotiana benthamiana* were infiltrated with0.4 O.D p35S-AKRP.1:GFP, as described in Billey et al., 2021. Two days post-infiltration (dpi), approximately 4g of transformed leaf segments were collected, rinsed with ddH2O, and ground with mortar and pestle in a 10-ml 1 × chloroplast isolation buffer (CIB):0.33M sorbitol,0.1M tris-Cl (pH 7.8), 5 mM MgCl2, 10 mM NaCl, and 2 mM EDTA supplemented with0.1% BSA. Here, all steps were performed on ice. Grounded leaves were passed through a 100-micron mesh filter into 15-ml falcon tubes and centrifuged at 1,000 × g for 3 min at 4oC. The supernatant were transferred to a sterile chilled 15-ml falcon tube and centrifuged for additional 7 min at 1,000 × *g*. The pellet was resuspended in 1 ml CIB with0.1% BSA. The mixture was gently loaded on a 40/80% percoll gradient (2.5 ml 80% and 5 ml 40%) in a 15-ml falcon tube. The gradient was centrifuged at 3,200 × *g* for 15 min at 4°C. Using a 1-ml cut tip, intact chloroplast from the gradient interface was transferred and suspended in 3 × volume CIB and centrifuged for 1 min at 1,700 × *g* table. Pelleted chloroplasts were resuspended in 50 ul CIB and aliquoted for live cell imaging or stored at −80°C for protein extraction.

For chlorophyll quantification, mature siliques from the wild-type Col-0 and *akrp*-3/+ plants were dissected, and wild-type green as well as mutant white *akrp*-3 seeds (100–200 seeds; see Figure 6) were collected in 1 ml dimethylformide (DMF) in triplicates: WT seeds: 200 seeds/replicate, Ht3: 200 seeds/replicate, Ht15: 133 seeds/replicate, and Ht22: 119 seeds/replicate. All the samples were incubated overnight at 4oC. Absorbance was measured at 664, 665, and 647 nm. The samples were diluted 3 × to fit the optical range and measured using a CLARIOstar microplate reader (BMG LABTECH, Ortenberg, Germany). The final amount (in ug/ml) of chlorophylls a and b, and total chlorophyll a + b was calculated according to Harris and Baulcombe (2015), as follows:

for chlorophyll a content (in μg/ml):

= (12 × A664.5) − (2.79 × A647),

for chlorophyll b content (in μg/ml):

= (20.78 × A647) − (4.88 × A664.5), and

for total chlorophyll content (in μg/ml): Chla + Chlb, later normalized to μg/ml per 100 ovules for each respective sample.

### Histochemical GUS Staining

Stable transformants were screened for promoter-GUS activity in two subsequent generations. Selected plant organs were incubated in a GUS staining buffer:0.2 M Na_2_HPO_4_,0.2 M NaH_2_PO_4_, 10 mM EDTA,,1% Triton X-100, 1 mM K_3_Fe(CN)_6_, and 2 mM X-Gluc, and stained for up to 48 h. Stained tissues were cleared from chlorophyll by bleaching in ethanol series (90, 70, and 50% (v/v)) prior to imaging.

### Microscopy

For mutant embryo phenotypic screening, seeds were cleared in a solution of chloral hydrate. Samples were observed under the DIC optics on a Nikon TE-2000 microscope. Images were processed in the NIS Elements software (LIM). Confocal images were taken on a Nikon Eclipse Ti confocal microscope equipped with a CSU-X1 spinning disk module and an Andor iXon3 EMCCD camera, as well as with a Zeiss LSM880 confocal microscope, and captured with the ZEN 2.3v software. The images were analyzed and assembled with the ImageJ/Fiji^3^,^4^, Adobe Photoshop CS6^5^, Ink-scape^6^, and NIS Elements (LIM) software. For colocalization study (based on a publication by Marcus and Raulet, 2013), a Zeiss LSM880 confocal microscope with these settings was used: 405-nm laser for excitation of GFP and 514-nm laser for excitation of YFP; emission was captured in a range of 481-508 nm for GFP and 552-561 nm for YFP.

### Statistics

All the statistical analyses were performed using the Chi square test at *p* < 0.01 or as specified in respective sections.

### RNA Extraction and qRT-PCR Analysis

Tissue samples were collected from the WT Col-0, *emb2036-3/*+ plants and complemented lines, and frozen in liquid nitrogen. The RNA was extracted using RNeasy Plant Mini Kit (Qiagen, Germany) and treated with RQ1 RNAse-free DNase I (Promega, Maryland, MD, United States). First-strand cDNA synthesis was conducted using a recombinant M-MLV reverse transcriptase (Promega, Maryland, MD, United States). Measurements of qRT-PCR were performed using a LightCycler 480 Instrument (Roche, Basel, Switzerland). Primers were designed to distinguish among splicing isoforms of AKRP. Translation initiation factor 1 alpha4 (AT560390G) was used as a reference gene. All the measurements were conducted in three biological and two technical replicates. A list of primers can be found in Supplementary Table S1.

### Cloning Strategies

All the PCR reactions were performed using Phusion and Q5 Polymerase (New England Biolabs, Hitchin, United Kingdom) according to the instructions of the manufacturer. Primer sequences are listed in Supplementary Table S1. For the AKRP promoter, 997 bp upstream of the start codon was cloned into the pENTR/D-TOPO vector (Invitrogen, Thermo Fisher, Germany) and recombined into the destination vector pKGWFS7 (Karimi et al., 2002) bearing two reporter genes, GUS and GFP. For full-length AKRP, an AKRP putative promoter (997 bp) and a coding sequence with/without stop codon were amplified from gDNA, cloned into the pENTR/D-TOPO vector, and recombined into the destination vector pB7FWG,0 (Karimi et al., 2002) bearing the eGFP marker. For complementation under the control of different promoters (pEC1.1, pDD1, p35S, and pLat52), CDS was amplified from cDNA and recombined into the pENTR/D-TOPO vector. Complementation constructs were created using either only the longer isoform (p35S, pEC1.1, pDD1) or both isoforms (pLat52) *via* Multisite Gateway technology (Invitrogen, Thermo Fisher Scientific, Germany). The pB7m34GW, 0 backbone, and eGFP marker were used. These vectors were transformed into *Arabidopsis* WT Col-0 and/or *emb2036-3/*+ plants by floral dipping (Clough and Bent, 1998).

## Data Availability Statement

Theoriginal contributions presented in the study are included in the article/Supplementary Material, further inquiries can be directed to the corresponding author/s.

## Author Contributions

SH and KK designed the experiments. KK, SH, VK, and JP conducted the experiments and analyzed the data with supervision by SH. SH, KK, and DH wrote the manuscript. All the authors read and approved the manuscript.

## Conflict of Interest

The authors declare that the research was conducted in the absence of any commercial or financial relationships that could be construed as a potential conflict of interest.

## Publisher’s Note

All claims expressed in this article are solely those of the authors and do not necessarily represent those of their affiliated organizations, or those of the publisher, the editors and the reviewers. Any product that may be evaluated in this article, or claim that may be made by its manufacturer, is not guaranteed or endorsed by the publisher.

## Footnotes

1
https://www.gabi-kat.de

2
https://seedgenes.org

3
http://imagej.net/

4
http://fiji.sc/Fiji

5
www.adobe.com

6
www.inkscape.org

## Supplementary Material

Supplementary Figure S1 Protein alignment of two AKRP splice variants. The short isoform differs by one amino acid substitution and 75 amino acid deletion comprising two ankyrin repeats at the C-terminus. 1. Alignment was performed with Clustal Omega using HMM model and visualized in Jalview v2.9.0b2.

Supplementary Figure S2 Profiling of AKRP promoter activity in various plant tissues. Detection of AKRP promoter activity in various tissues: cotyledons and stipules of 7-day old seedlings **(a,b)**, inflorescence **(c)**, uninucleate microspores **(d)**, bicellular pollen **(e)**, mature pollen **(f)**, immature **(g)**, and mature **(h)** ovules, and globular to cotyledon stage seeds and embryos **(i–k)**. Scale bar = 20 μm.

Supplementary Figure S3 Gametophytic viability and pollen tube assays. **(a,b)** Alexander staining and quantification of pollen viability, (*n* = 664). **(c)** Comparative assessment of *in vitro* pollen germination. **(d)** Aniline blue staining of self-pollinated pistils 24 h after pollination revealed severe targeting defect (*n* = 953); this panel supports Figure 2. **(e)** Estimation of *in vitro* and semi-*in vivo* pollen tube length. **(f)** Confocal imaging of egg cell (EC1.1)-specific pEC1.1-H2B-mRFP (*n* = 2,511), vegetative cell-specific pLat52-GUS (*n* = 3,233) and a double marker for sperm cell (SC) pHTR10-HTR10:RFP (*n* = 4,342) and vegetative cell (VC) pLat52-GFP (*n* = 2,313). **(g)** Quantification of marker expression in *akrp-3/*+ and segregated WT plants. AC, antipodal cell expression of pDD1-AKRP.1::GFP.

Supplementary Figure S4 The *akrp*-3 transmission test and blue dot assay. **(a)** Segregation of self-fertilized *akrp-3/*+ on sulfadiazine antibiotic. **(b)** Transmission test of the *akrp-3* allele after reciprocal crosses. TE = transmission efficiency (Wt/Het × 100), χ^2^ test: not significant at *p* < 0.01. **(c)** Blue dot assay to assess ovule attractivity following pollination with pLAT52-GUS-expressing pollen suggests a dramatic decrease in *akrp*-3 pollen tube attraction efficiency 24h after pollination (*n* = 824). This panel supports Figure 2. χ^2^test, significant at *p* < 0.01. Scale bar = 50 μm. **(d)** Induction of aborted ovule phenotype through an *akrp*-3 female after reciprocal crosses.

Supplementary Figure S5 Cell type-specific complementation of *akrp-3* mutation. **(a,b)** Complementation using a p35S promoter (35S-AKRP.1:GFP) yielded rescued homozygous plants already in the first generation. The complementation was not absolute, because some lines retained some levels of embryonic defects; **(c,d)** Chlorotic cotyledons that recovered in newly emerged true leaves and **(e)** lesions in rosette leaves. **(f)** Complementation with an egg cell-specific promoter (pEC1.1) was not sufficient to rescue *akrp*-3 fertilization or embryo-lethal phenotype (*n* = 11). **(g)** Complementation under an antipode-specific pDD1 promoter weakly rescued the embryo-lethal *akrp-3* phenotype (class labeled as “other,” phenotypically similar to Figure 6B “other” category, *n* = 5).

Supplementary Figure S6 AKRP.1 plastid co-localization in mature pollen. Analysis of double heterozygous Lat52-AKRP.1:GFP and pBINU-CHYA(K) marker in mature pollen by independent GFP and YFP channel excitation with Argon 488 laser in a Zeiss confocal microscope. Intensity values were plotted using the Prism software.

Supplementary Table S1 List of primers used in this study.
