# Supplementary figures and images for: A Plastid-Bound Ankyrin Repeat Protein Controls Gametophyte and Early Embryo Development in Arabidopsis thaliana
Source: Front Plant Sci. 2022 Mar 8;13:767339. doi: 10.3389/fpls.2022.767339 (PMC8958021; doi:10.3389/fpls.2022.767339)

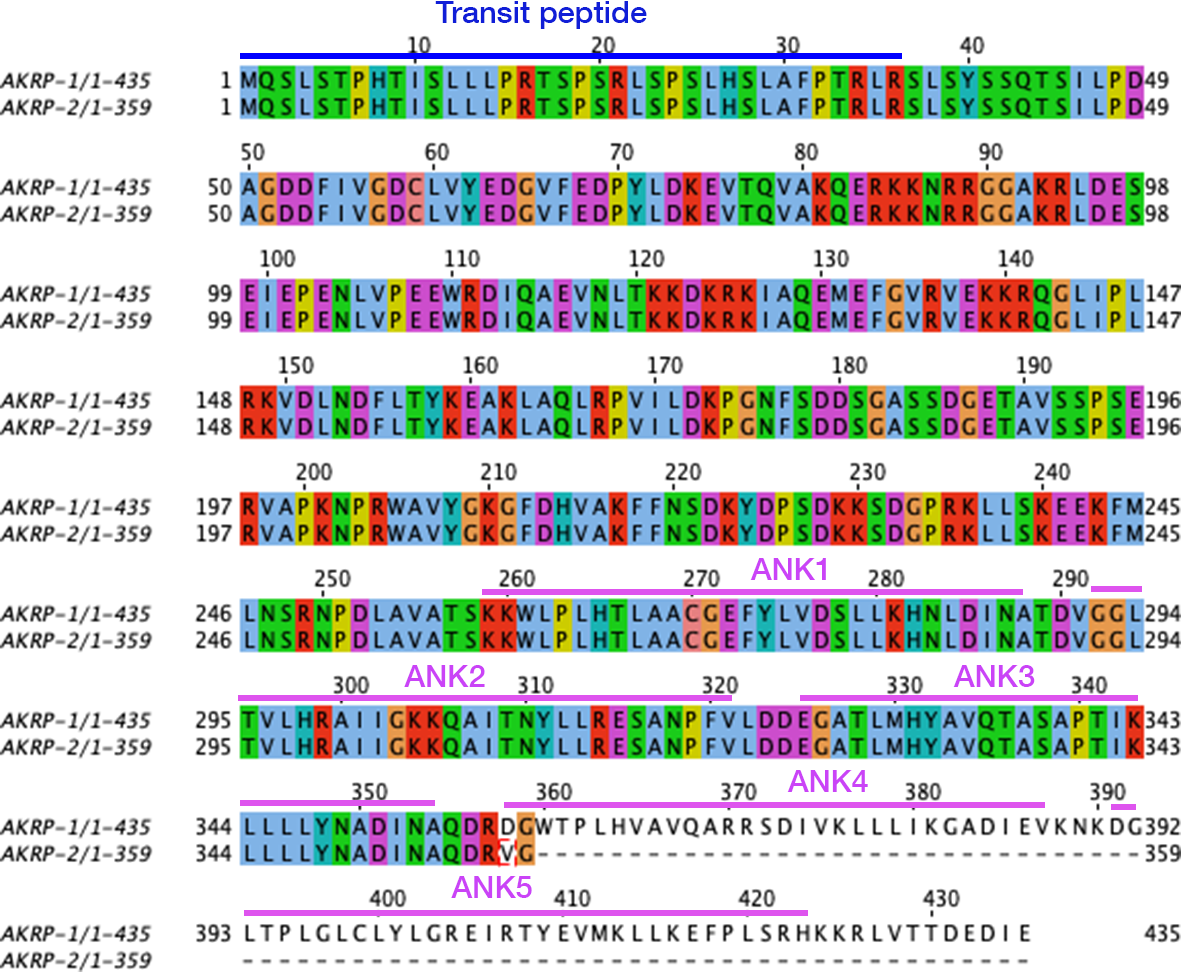

Supplement: Supplementary Figure S1 — Protein alignment of two AKRP splice variants. The short isoform differs by one amino acid substitution and 75 amino acid deletion comprising two ankyrin repeats at the C-terminus. 1. Alignment was performed with Clustal Omega using HMM model and visualized in Jalview v2.9.0b2. [file Image_1.tif]

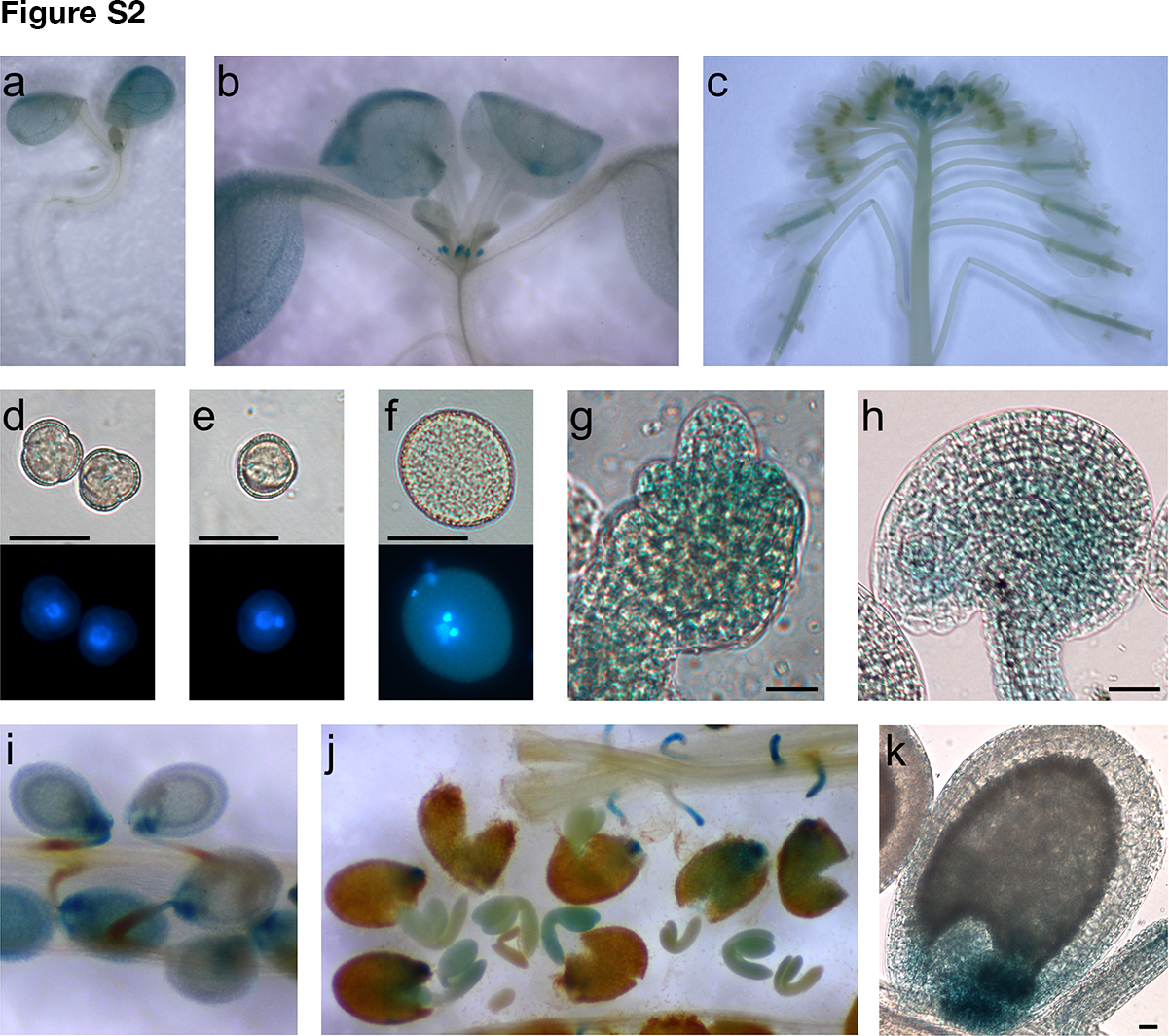

Supplement: Supplementary Figure S2 — Profiling of AKRP promoter activity in various plant tissues. Detection of AKRP promoter activity in various tissues: cotyledons and stipules of 7-day old seedlings (a,b), inflorescence (c), uninucleate microspores (d), bicellular pollen (e), mature pollen (f), immature (g), and mature (h) ovules, and globular to cotyledon stage seeds and embryos (i–k). Scale bar = 20 μm. [file Image_2.TIF]

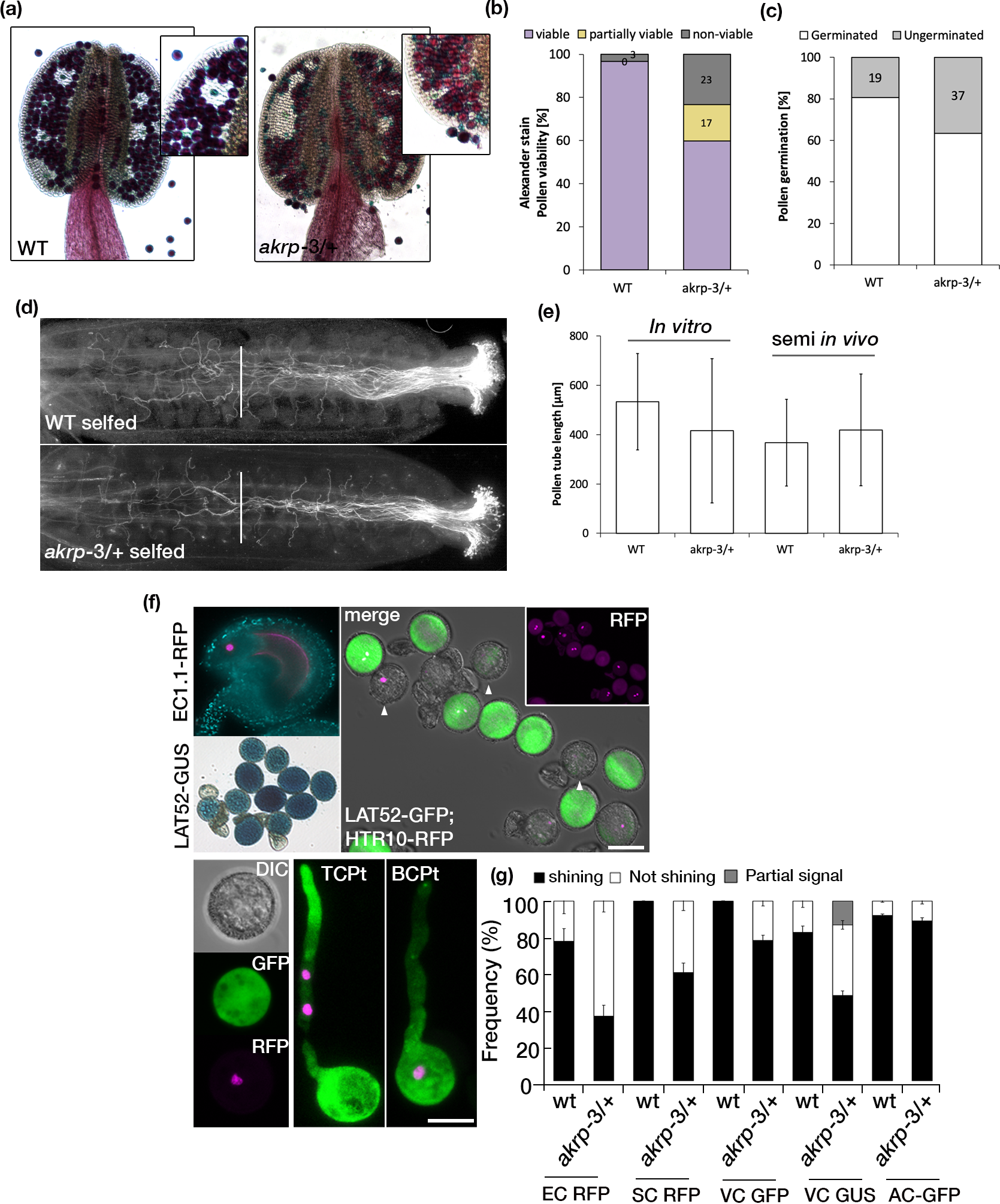

Supplement: Supplementary Figure S3 — Gametophytic viability and pollen tube assays. (a,b) Alexander staining and quantification of pollen viability, (n = 664). (c) Comparative assessment of in vitro pollen germination. (d) Aniline blue staining of self-pollinated pistils 24 h after pollination revealed severe targeting defect (n = 953); this panel supports Figure 2. (e) Estimation of in vitro and semi-in vivo pollen tube length. (f) Confocal imaging of egg cell (EC1.1)-specific pEC1.1-H2B-mRFP (n = 2,511), vegetative cell-specific pLat52-GUS (n = 3,233) and a double marker for sperm cell (SC) pHTR10-HTR10:RFP (n = 4,342) and vegetative cell (VC) pLat52-GFP (n = 2,313). (g) Quantification of marker expression in akrp-3/+ and segregated WT plants. AC, antipodal cell expression of pDD1-AKRP.1::GFP. [file Image_3.tif]

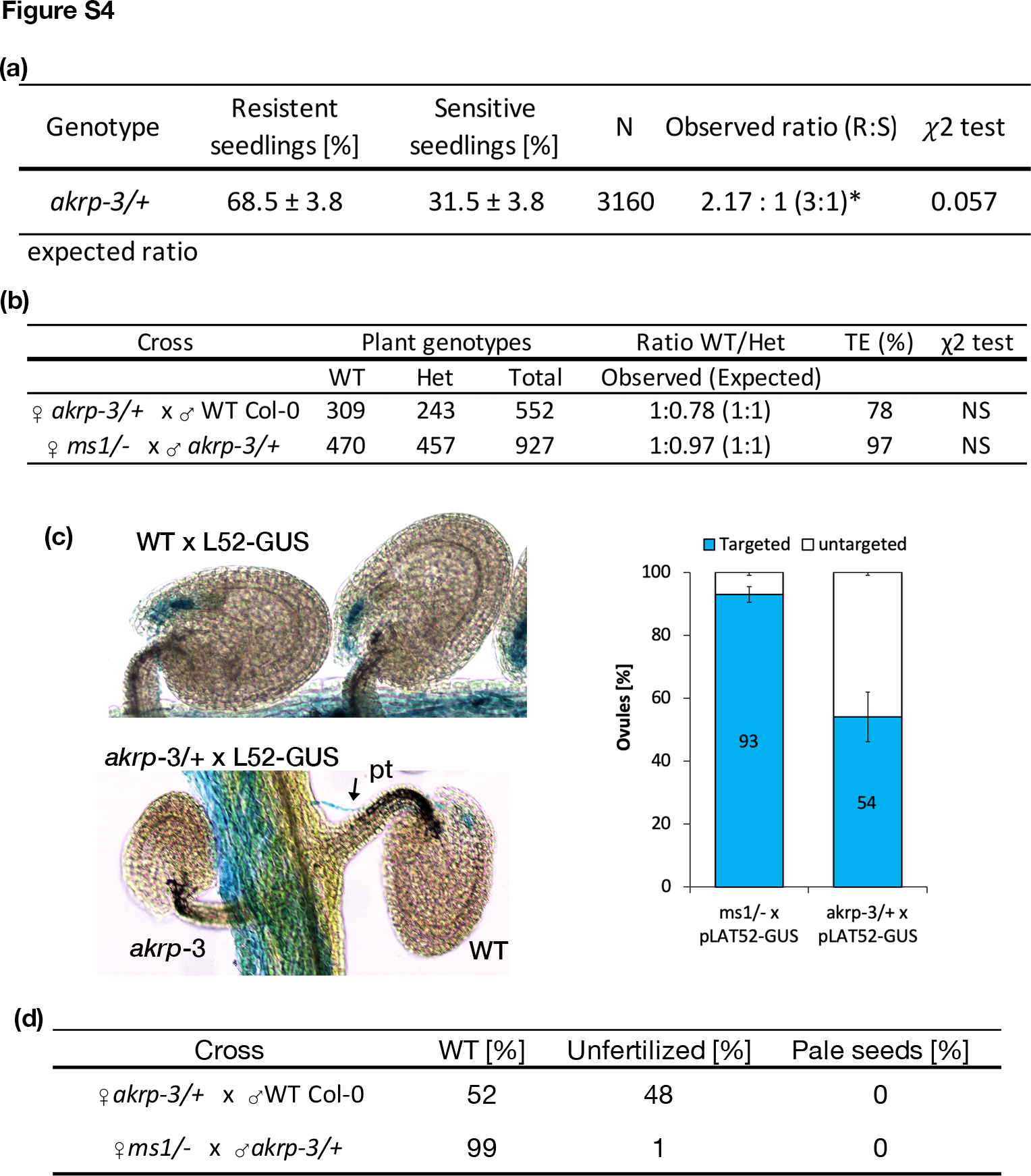

Supplement: Supplementary Figure S4 — The akrp-3 transmission test and blue dot assay. (a) Segregation of self-fertilized akrp-3/+ on sulfadiazine antibiotic. (b) Transmission test of the akrp-3 allele after reciprocal crosses. TE = transmission efficiency (Wt/Het × 100), χ2 test: not significant at p < 0.01. (c) Blue dot assay to assess ovule attractivity following pollination with pLAT52-GUS-expressing pollen suggests a dramatic decrease in akrp-3 pollen tube attraction efficiency 24h after pollination (n = 824). This panel supports Figure 2. χ2test, significant at p < 0.01. Scale bar = 50 μm. (d) Induction of aborted ovule phenotype through an akrp-3 female after reciprocal crosses. [file Image_4.TIF]

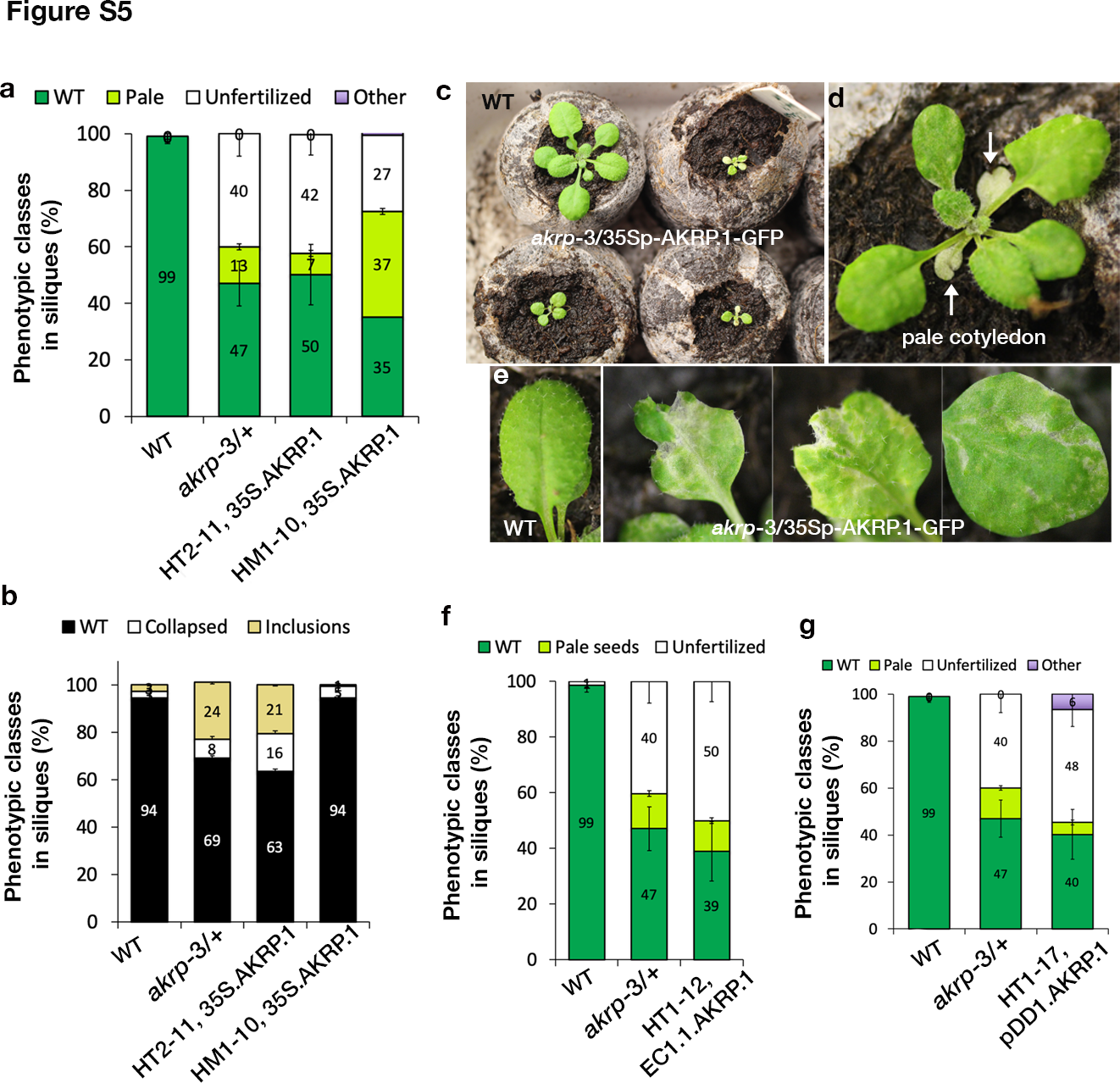

Supplement: Supplementary Figure S5 — Cell type-specific complementation of akrp-3 mutation. (a,b) Complementation using a p35S promoter (35S-AKRP.1:GFP) yielded rescued homozygous plants already in the first generation. The complementation was not absolute, because some lines retained some levels of embryonic defects; (c,d) Chlorotic cotyledons that recovered in newly emerged true leaves and (e) lesions in rosette leaves. (f) Complementation with an egg cell-specific promoter (pEC1.1) was not sufficient to rescue akrp-3 fertilization or embryo-lethal phenotype (n = 11). (g) Complementation under an antipode-specific pDD1 promoter weakly rescued the embryo-lethal akrp-3 phenotype (class labeled as “other,” phenotypically similar to Figure 6B “other” category, n = 5). [file Image_5.TIF]

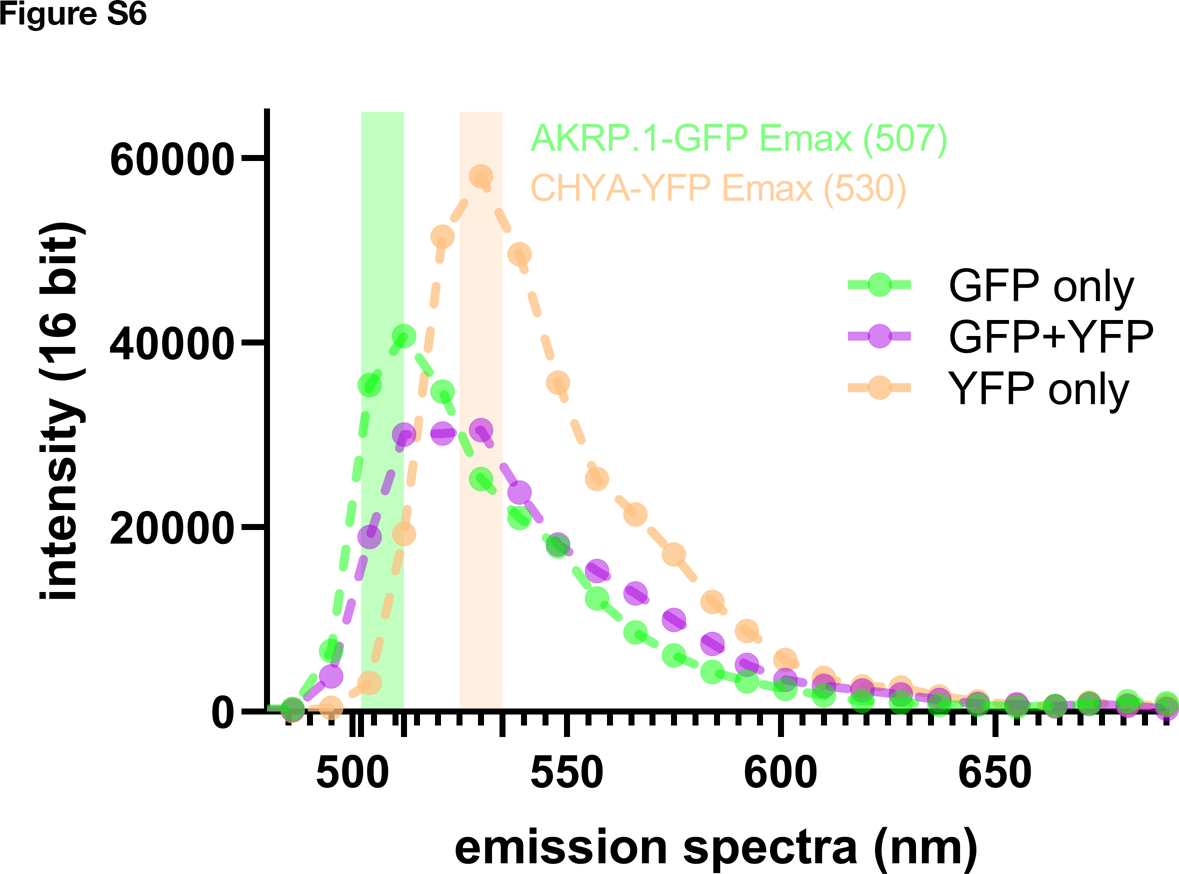

Supplement: Supplementary Figure S6 — AKRP.1 plastid co-localization in mature pollen. Analysis of double heterozygous Lat52-AKRP.1:GFP and pBINU-CHYA(K) marker in mature pollen by independent GFP and YFP channel excitation with Argon 488 laser in a Zeiss confocal microscope. Intensity values were plotted using the Prism software. [file Image_6.TIF]
